# Effect of advanced care on psychological condition in patients with chronic renal failure undergoing hemodialysis: A protocol of a systematic review

**DOI:** 10.1097/MD.0000000000014738

**Published:** 2019-03-08

**Authors:** Yun Guan, Yu-xia He

**Affiliations:** a Department of Nursing; b Department of Nephrology, Yanan University Affiliated Hospital, Yan’an, China.

**Keywords:** advanced care, chronic renal failure, effect, hemodialysis, randomized controlled trial

## Abstract

**Background:**

The protocol of this systematic review will be proposed for assessing the effects of advanced care (AC) on psychological condition in patients with chronic renal failure (CRF) undergoing hemodialysis.

**Methods:**

We will search the following electronic databases: MEDLINE, EMBASE, Cochrane Central Register of Controlled Trials (CENTRAL), Web of Science, Chinese Biomedical Literature Database, and China National Knowledge Infrastructure from inception to the January 30, 2019. Any randomized controlled trials (RCTs) for assessing the effects of AC on psychological condition in patients with CRF undergoing hemodialysis will be fully considered. The methodological quality will be assessed by using Cochrane risk of bias tool. Two independent reviewers will perform the study selection, data extraction, and methodological quality assessment. A third reviewer will be invited to judge the disagreements between 2 reviewers by discussion.

**Results:**

The protocol of this proposed systematic review will compare the effects of AC on psychological condition in patients with CRF undergoing hemodialysis. The outcomes will comprise of depression. The secondary outcome includes anxiety, health related quality of life, and any adverse events.

**Conclusion:**

The findings of this systematic review will summarize the latest evidence of AC on psychological condition in patients with CRF undergoing hemodialysis.

**Ethics and dissemination:**

All data used in this systematic review will be collected from previous published clinical studies. Thus, no ethic approval is required for this study. The findings of this study will be published at a peer-reviewed journal.

**PROSPERO registration number:**

PROSPERO CRD42019122275.

## 1 Introduction

Chronic renal failure (CRF) is an irreversible deterioration disorder for renal function.^[1,2]^ It is often characterized by a variety of disorders that involve many organs, such as heart, bone, blood vessels, and peripheral nerves.^[3–6]^ Hemodialysis is one of the most effective managements for patients with CRF, which can significantly enhance their renal function and life quality and prolongs survival time.^[7–9]^ However, patients with CRF undergoing hemodialysis often suffer from psychological conditions, such as depression, anxiety, and poor quality of life.^[10–14]^

A variety of clinical studies have reported that advanced care (AC) can be used to manage psychological conditions for patients with CRF under hemodialysis.^[15–31]^ However, up to the present, no systematic review has addressed to investigate the effectiveness and safety of AC for psychological condition in patients with CRF undergoing hemodialysis. Therefore, in the present systematic review, we will firstly assess the effectiveness and safety of AC for psychological condition, including depression and anxiety in patients with CRF receiving hemodialysis.

## 2 Methods

### 2.1 Criteria for included studies

#### 2.1.1 Study types

This proposed study will only consider randomized controlled trials (RCTs) that have evaluated all form of AC on psychological condition in patients with CRF undergoing hemodialysis. However, the other studies except the RCTs will not be considered, such as non-clinical studies, non-controlled studies, non-RCTs, and quasi-RCTs.

#### 2.1.2 Participants

All patients with CRF accepting hemodialysis are clinically diagnose as having psychological conditions, including depression and anxiety will be fully considered regardless the race, sex, and age.

#### 2.1.3 Interventions

The patients in the experimental group have received any forms of AC. The patients in the control group can be treated with any interventions, except the AC.

#### 2.1.4 Outcomes

The primary outcome includes depression, as assessed by Hamilton Depression Rating Scale or any others. The secondary outcomes consist of anxiety, as measured by Hamilton Anxiety Rating Scale or any others; health related quality of life, as assessed by 36-Item Short Form Health Survey or any others; as well as any adverse events.

### 2.2 Strategy of literature searches

The potential studies will be searched from the following databases from inception to the January 30, 2019 without any language limitations: MEDLINE, EMBASE, Cochrane Central Register of Controlled Trials (CENTRAL), Web of Science, Chinese Biomedical Literature Database, and China National Knowledge Infrastructure. The RCTs on evaluating the effects of AC for psychological condition in patients with CRF undergoing hemodialysis will be included in this systematic review. In addition, the websites of clinical registry, and reference lists of eligible studies will also be searched. We have provided the sample of detailed search strategy for database CENTRAL in Table 1. The identical search strategies for other databases will be built and applied.

**Table 1:**
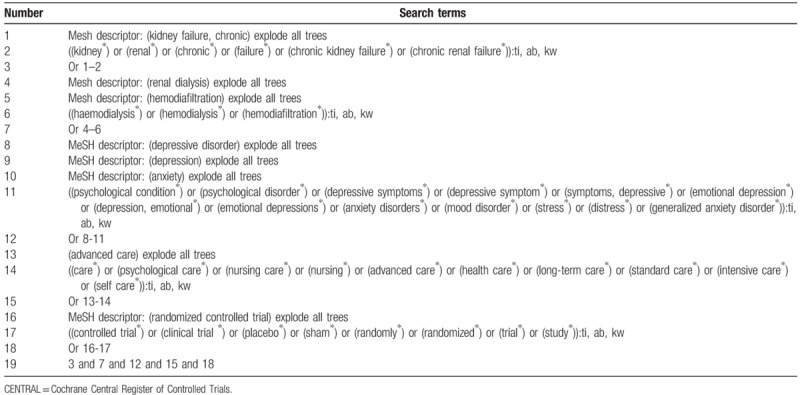
Search strategy applied in CENTRAL database.

### 2.3 Data collection

#### 2.3.1 Study selection

**Figure 1:**
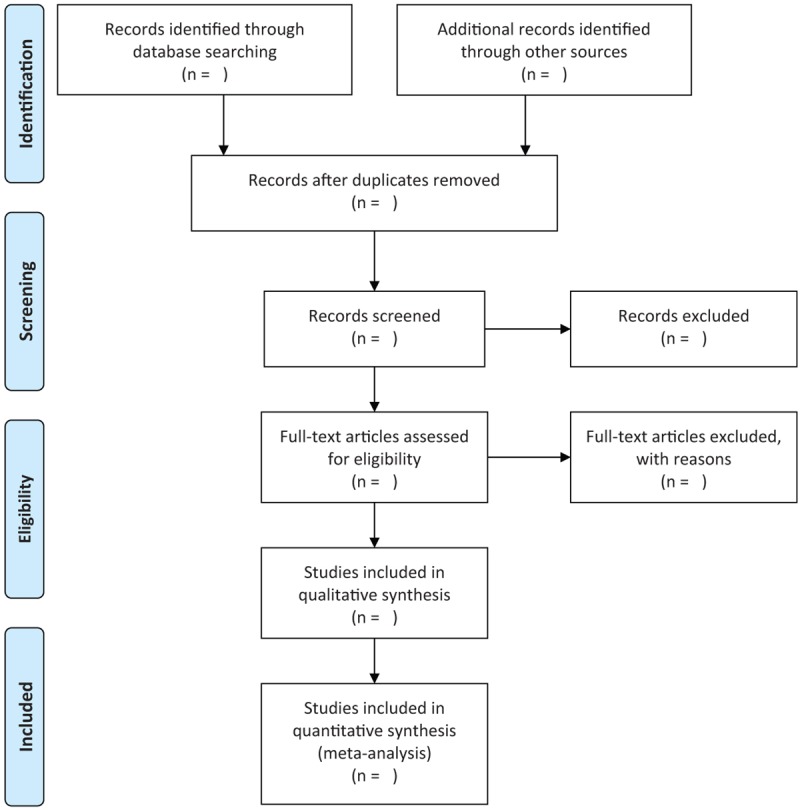
Flowchart of study selection.

Two reviewers will independently screen the titles and abstracts, as well as read full-texts if studies meet primary inclusion criteria according to the pre-defined eligibility criteria. Any divergences regarding the study selection between the 2 reviewers will be consulted with a third reviewer. The procedures of study selection are shown in Fig. 1.

#### 2.3.2 Data extraction

For all included studies, 2 reviewers will independently extract data by using a predefined data extraction form. The extracted information includes study characteristics (such as first author, publication year, title, journal, location), patient characteristics (such as diagnostic criteria, age, sex, number of patients), study methods (such as details of randomization, concealment, blinding, and any other bias), interventions (such as dosage, frequency, duration), and outcomes (such as primary, secondary, and any other outcome measurements). Any disagreements between 2 reviewers will be solved by discussion with a third reviewer.

#### 2.3.3 Dealing with missing data

If any insufficient or missing data will be identified, we will contact the primary authors to inquire them. If those data cannot be achieved, we will just analyze the available data, and will discuss it.

### 2.4 Methodological quality assessment for included studies

To evaluate the methodological quality for each included trial, 2 reviewers independently assess the risk of bias by using Cochrane Collaboration Tool. It includes 7 items, and each item is classified as high, unclear, and low risk of bias.

Any divisions will be solved with a third reviewer through discussion.

### 2.5 Statistical analysis

We will use ReMan 5.3 software to carry out the statistical analysis. All continuous data will be expressed as mean difference or standardized mean difference and 95% confidence intervals (CIs), and all dichotomous will be expressed as risk ratio and 95% CIs. *P* < .05 is regarded as having statistically significance.

Heterogeneity among eligible studies will be identified by *I*^2^ test. If *I*^2^ ≤50%, a minor heterogeneity is considered, fixed-effect model will used to pool the data and meta-analysis will be conducted. If *I*^2^ >50%, substantial heterogeneity is regarded, and a random-effect model will be used to pool the data, and meta-analysis will conducted or not according to the heterogeneity after subgroup analysis. If there is still substantial heterogeneity after subgroup analysis, data will not be pooled, and meta-analysis will not be conducted. However, a narrative summary will be reported.

Subgroup analysis will be performed in accordance with the different treatments, controls, and outcome measurements to identify any possible reasons that may result in the significant heterogeneity. Sensitivity analysis will also be carried out to check the robustness of pooled outcomes by taking away low quality studies. If >10 RCTs are entered in this study, funnel plot^[32]^ and Egg regression^[33]^ will also be operated to identify if there is reporting bias.

## 3 Discussion

CRF is one of the severest renal diseases, and often result in high mortality and morbidity.^[1,2]^ Hemodialysis is reported to effectively treat such condition.^[7,8]^ However, patients with CRF accepting hemodialysis often experience serious psychological conditions, including depression and anxiety. Up to now, no systematic review has investigated effectiveness and safety of AC for the management of psychological conditions in patients with CRF undergoing hemodialysis, although a lot of clinical studies have reported that AC can manage these psychological disorders effectively.^[15–31]^

This study will firstly and systematically assess the effectiveness and safety of AC for psychological conditions in patients with CRF accepting hemodialysis. Its results will provide rigorous summary evidence and will inform our understanding of AC for psychological conditions in patients with CRF undergoing hemodialysis across all published RCTs.

## Acknowledgments

Yan’an Specialized Project for Transformation and Promotion of Achievements (2018CGZH-15). The funder had no role in this study.

## Author contributions

**Conceptualization:** Yun Guan, Yu-xia He.

**Data curation:** Yun Guan, Yu-xia He.

**Formal analysis:** Yun Guan, Yu-xia He.

**Funding acquisition:** Yun Guan.

**Investigation:** Yu-xia He.

**Methodology:** Yun Guan.

**Project administration:** Yun Guan, Yu-xia He.

**Resources:** Yun Guan, Yu-xia He.

**Software:** Yun Guan, Yu-xia He.

**Supervision:** Yu-xia He.

**Validation:** Yun Guan.

**Visualization:** Yun Guan, Yu-xia He.

**Writing – original draft:** Yun Guan, Yu-xia He.

**Writing – review & editing:** Yun Guan, Yu-xia He.
